# Notes and News

**Published:** 1887-12-24

**Authors:** 


### Notes and News.

Collected and Communicated.

Dr. Booth has resigned his appointment as house physician at the Seamen's Hospital, Greenwich. Dr. Booth entered the service of the society as house surgeon in 1883, and was appointed house physician in 1884.

The Governors of Carlisle Infirmary have resolved to carry out a scheme of drainage, heating, and ventilation which will cost between £500 and £600.

A fancy fair on behalf of the National Children's Hospital, Dublin, was held on Tuesday, Wednesday, and Thursday last. Her Serene Highness Princess Edward of Saxe-Weimar opened the bazaar. Besides the ordinary attractions of a bazaar, there were amateur theatricals. tableaux vivants, and other inducements to visitors and purchasers.

It is proposed to provide Chelmsford with a hospital for infectious diseases, but there is some opposition to the scheme, as the price asked for the site selected—£480 for three acres —is considered excessive by some members of the Local Board of Health, one member of which points out that the town already owns a piece of ground suitable for the erection of a hospital, and therefore does not need to buy another.

The Grimsby Hospital is wise enough to accept subscriptions in other forms than money. At the board meeting held at the end of November the committee had to acknowledge, as well as a donation of £500 from Mr. H. J. Veal, for the purpose of adding an "emergency wing" to the hospital, several gifts of fish, ice, and meat for soup. It is highly probable that our London hospitals would not resent the offer of similar gifts, and we commend this form of charity to London tradesmen. They would not greatly miss what they gave in this way, and such assistance would be valued by hospital authorities.

Mrs. Katherine S. Macquoid makes an appeal through the newspapers on behalf of the Cheyne Hospital for Incurable Children. The work of the hospital has been hitherto carried on in two small houses, 45 and 47, Cheyne Walk, Chelsea. Those buildings are not only old and decayed, but are altogether too small to receive the numerous urgent cases which seek admittance. A site facing the river, and close to old Chelsea Church, has been secured for a new hospital, and Mrs. Macquoid urgently appeals to the charitable public for help, so that the new buildings may be begun with the new year.

The sum contributed up to date towards the Aberdeen Royal Infirmary Extension Fund amounts to £26,108 15*s.* 6*d.*

It was stated last week that Dr. W. E. Steavenson had been appointed to serve on the Nurses' Registration Committee of the Hospitals Association. Dr. Steavenson asks us to state that such is not the case.

The foundation-stone and four corner-stones of a Jubilee Children's Hospital were laid at Gateshead on the 9th inst. The site of the new hospital commands a beautiful view of the river Tyne and Ravensworth Woods, and has generously been given to the committee by Lady Northbourne.

A complimentary concert to the lady collectors of the Hospital Saturday Fund in the central districts, was given at the Memorial Hall, Farringdon Street, on the 10th inst., by the Poplar Musical Union. Through the efforts of these ladies many thousands of pounds have been added to the fund during the past thirteen years. Through the generosity of Miss Grace Hawthorne, an eight days' benefit at the Princess's Theatre is being given to the fund from the 16th to the 23rd inst. inclusive.

It affords us great pleasure to announce that we have made arrangements to publish with our next issue *The Hospital Almanac for* 1888, prepared by Mr. F. C. Carr-Gomm, Chairman of the London Hospital, which will contain a sketch of St. Thomas's Hospital, and full particulars of all the metropolitan hospitals; the name, address, date of foundation, governing bodies, resident management and nursing staff, visiting and resident medical officers, the total and average number of beds, the average cost per in-patient per week, the number of in and out patients, the sources of income, the qualifications for governorship, the endowed beds and their cost; with particulars of the medical colleges, the samaritan funds, convalescent homes, nursing schools, and many other facts of general interest to the public, the profession, and hospital officials. This Almanac cannot fail to be of value and interest, and those who desire to secure copies should at once send their orders to the publisher. Each regular subscriber will be presented with a free copy of the Almanac; the price to others will be 6*d.* for single copies, or 5*s.* per dozen.

We call attention with great pleasure to the communication from Mr. Gladstone published in another column. The subject of scarlet fever convalescents is one well calculated to attract and fix the attention of all classes in the community, for there are few families which have not suffered from the ravages of this fell disease. Even now the epidemic of scarlet fever in London has only abated, and is, we fear, by no means at an end. In these circumstances it concerns the public to know and to understand that the convalescent stage of scarlet fever is the most dangerous to the patient as well as to others. The convalescent scarlet fever patient is a pepper-box of infection, and in this condition is particularly susceptible to cold, which may lay the seeds of an organic lesion that may prove fatal many years afterwards. Indeed, experience shows that it is not the most severe cases of scarlet fever which usually result in the greatest mischief. Such cases are regarded by the friends as serious, and they take the utmost care to prevent all after mischief. In the mild cases, and especially in childhood, when children are sometimes literally in bed one day and up two days afterwards, there is the greater danger, because it is so difficult to make parents understand that the children are still in a condition necessitating the greatest care if after effects of a serious character are to be avoided. Thus convalescence being the stage of scarlet fever which demands the utmost care and precaution, Mr. Gladstone's article will no doubt be welcomed by many, and read by all.

THE following vacancies are announced this week, the amount of salary in each case being given after the name of the institution :—

**Resident Medical Officers.**

Scarborough Hospital and Dispensary. House Surgeon.—£80.
Addenbrooke's Hospital, Cambridge. Resident House Physician. —£65.
Birmingham General Hospital. Assistant House Surgeon.
Seamen's Hospital, Greenwich. House Physician —£75.
Dorset County Hospital, Dorchester. House Surgeon and Secretary.—£70 as House Surgeon, and £10 as Secretary.
Seamen's Hospital Society, Greenwich. House Physician.—£75, board, rooms, and attendance.

**Non-resident Medical Officers.**

Essex and Colchester Hospital. Physician.—£200.
Lymington Union. Medical and Vaccination Officer.—£65, with the usual extra medical and vaccination fees.
Wigan Union. Medical and Vaccination Officer.—£130, with the usual medical and vaccination fees.

**Matron.**

Tewkesbury Hospital. Matron Nurse. 18 beds.—£40 for first year, £45 and £50 for each succeeding year.

**Nurses.**

Salop Infirmary. Head Nurse, thoroughly experienced in Medical and Surgical Nursing
Whitchurch Hospital. Trained Nurse under Matron of Cottage Hospital, work principally out of hospital.—Salary, £25.
Royal Hospital, Portsmouth. Thoroughly trained Head Nurse for Male Surgical Wards.—£25, with uniform.
City of London Hospital for Diseases of the Chest. Medical Nurse.—£20 to £24, with uniform.
Nurses' Institute, Canterbury. Three trained Nurses.
Children's Hospital, Bristol. Two Nurses for Medical Wards.
Rotherham Hospital and Dispensary. Nurse for Males' Surgical Ward. Age 28 to 30.—£23, uniform and washing.
Home for Trained Nurses, Frome. Trained Nurse for Private and District Work.—£20 to £25, with uniform.
Weston-super-Mare. Trained Night Nurse for Male and Female Wards.—£25.
Rotherham Hospital. Trained Nurse, Surgical and Medical.
Bootle Borough Hospital. One Special and one Nurse Probationer.

**Probationer.**

Torbay Hospital, Torquay, Devon. Working Probationer.

THE Council of the Darlington Hospital have decided to add a convalescent ward to the Fever Hospital. In view of this, the Matron, Miss Charlton, has asked the inhabitants for gifts of pictures, screens, and any other articles suitable for decorating the ward, and making it as light and cheerful as the environment of convalescents ought to be. As Miss Charlton has won the affection of the patients under her charge, as well as the esteem of the hospital committee, an appeal from her should not fail of success.

THE Committee of Management of the National Eye and Ear Hospital, Dublin, met on Thursday, the 8th inst., when the treasurer drew attention to the fact that, in spite of the utmost economy in management, the balance at the bank to the credit of the hospital was overdrawn to the amount of £40, and appealed to the public to enable the institution to clear off this debt before the end of the year.

MR. HENRY TOWNSHEND, of Caldecote Hall, Nuneaton, presided at the annual meeting of the subscribers and friends of the Coventry and Warwickshire Hospital on Saturday afternoon. The Mayor of Coventry (Mr. Alderman Tomson) the Revs. F. M. Beaumont and J. Butler, Drs. Milner Moon, Haig, and Miller, and Messrs. A. Seymour (secretary), J. Sutton, W. Pulley, and many others also attended. The report showed that the income of the institution during the year had been £3,209 7*s*. 2*d*. ; as against this, the expenditure amounted to £2,575 19*s*. 4*d*., leaving a balance of income over expenditure of £435 7*s*. 10*d*. This very satisfactory surplus enabled the committee to discharge a debt of £440 6*s*. 9*d*., and to commence the new financial year with a credit balance of £13 1*s*. 1*d*. The report went on to state that in every other respect the history of the year's operations was equally satisfactory.

THE second half-yearly collection of workpeople's subscriptions on behalf of the Warrington Infirmary amounts to £182 4*s*. 7*d*., and includes contributions from all the important factories in the district.

A SPECIAL service was held in the chapel of King's Hospital, Blackhall Place, Dublin, on the 11th inst., in celebration of the reopening of the chapel after extensive alterations and improvements. The Archbishop of Dublin preached an appropriate sermon.

WE propose to commence to devote a portion of our space every week from January 1st onwards to subjects of interest to all engaged in nursing. This department of the paper will be entitled "The Mirror of Nursing," and we shall be glad to receive notes on cases taken by nurses, announcements of vacancies and appointments as matrons, lady superintendents, and sisters, the results of examinations with the prize-winners at the various training schools, etc., and any questions upon subjects relating to nursing about which information may be desired. All matter for this column should be addressed to the Editor, The Lodge, Porchester Square, W.

ON Wednesday a sale of articles, including clothing, made by the working parties and invalids, took place at the Brownlow Road Nursing Home for permanently invalid ladies. Mrs. Sykes, Miss Morris, Miss Ruddock, and the Misses Bedford presided at the stalls, and the sum of £32 was realised. The enlarged part of the house is now ready for occupation, but owing to the scarcity of funds it cannot as yet be filled. The domestic arrangements having been altered, there is now a room which might easily be adapted for cancer cases, and would be so utilised if the management felt sure of an increased number of subscribers.

ADMIRAL INGLEFIELD writes to the *Morning Post* : "It is satisfactory to be able to report that the Lady Strangford Hospital at Port Said has now been in good working order for some months, and has been of service to many sufferers who have been landed there from steamers passing through the Suez Canal. Judging from the number already received, more than two hundred patients may be expected in a year, and of these many are likely to be serious cases, chiefly sailors. The committee have still to make up a deficiency of £1,700, and about £500 more will be needed to build a separate ward for isolated cases."

THE Annual Conference of Delegates from Boards of Guardians in England and Wales was held in Exeter Hall on Wednesday, December 7th, when an interesting discussion took place on the boarding-out of pauper children. The opening paper was read by Miss Joanna Hill, of Chipping Norton, who stated that the number of children boarded out in England and Wales amounted to 1,177 under 71 committees, and 1,863 under the relieving officers of 148 unions. She expressed high approbation of the boarding-out system, as being based on the order of nature—an opinion in which most of the guardians present agreed. Mr. T. H. Gordon, of Nottingham, stated that though by the system employed in his union it cost 4*s*. 6*d*. a-week to board each child out, as against 3*s*. 6*d*. for each in the workhouse, he and his fellow-guardians preferred the larger expenditure, as giving the children a better chance of growing up as useful citizens. Mr. R. J. Takin pointed out that by the boarding-out system the children got broader views of the world, and escaped the workhouse taint. Mrs. H. Kingsley advised a more constant supervision of the children by lady visitors, who could inquire into their comfort better than men. It is satisfactory to see the strong spirit of humanity evinced by the Conference, and to know that a large proportion of the poor-law guardians are honestly anxious to do all they can for the welfare of the children entrusted to their charge.

A largely-attended meeting of the newly-formed University Medical Society of Aberdeen was held last week in Marischal College, when Mr. Arthur Keith, the president, delivered an address on "Medical Etiquette."

A humorous "special constable" suggests, through the columns of the *Globe*, that as the "specials" are to be called out on Christmas Day, he thinks they would be justified in going round to the rich inhabitants and asking for Christmas boxes, the amount of which might afterwards be handed to some hospital.

The Leeds Workpeople's Hospital Fund amounts to £123 16*s.* 2*d.* A subscription of this amount shows the growing appreciation among the working classes of the fact that they benefit in a special manner by the existence of hospitals, and makes it clear that they are desirous of helping to support them.

Messrs. R. Ruthven Pym and the Rev. Benjamin Waugh, the Treasurer and Secretary of the Society for the Prevention of Cruelty to Children, have written to the newspapers, giving the statistics of their valuable work, and appealing for subscriptions to aid them in it. During the last three years they have dealt with 762 more or less cruel people, for the most part by moral influence. In 132 cases of terrible cruelty they have resorted to the law, and have obtained 120 convictions. In the 762 cases they have dealt with, 333 were cases of injury from assault, 81 of starvation, 130 of dangerous neglect, 32 of desertion, 70 of exposure to excite sympathy in the streets, and 116 of miscellaneous wrongs. Lest it should be thought that the action of the Society tends to lessen the authority of parents, it should be stated that they do not interfere with mere parental indiscretion, but only with deeds of cruelty which render child-life unendurable, 25 cases ending with the victims' death. When punishment is inflicted it is always followed by indirect but real supervision. The office of the Society is at 7, Harpur Street, W.C., where subscriptions will be gratefully received.

We have of late heard so much about the "unemployed" that was little to their credit, that we have been inclined to think that they were merely idle loafers, who did not suffer half so much from hunger and cold as they pretended, and at any rate did not desire to escape distress by means of honest work. Plenty of such there are, but they only trade upon a real misery that is everywhere present. If there was no genuine destitution, it would be impossible for the sham sufferers to gain a hearing for their pretended woes. Unfortunately, they discredit the cause they profit by, and charity, often deceived, is apt to withdraw in disgust. But that there is real poverty, and even starvation, around us, a case that came up before Mr. de Rutzen sufficiently proves. Two young men were seen begging in Kilburn by a detective, who was just enough to inquire into the truth of the tale they told him. It was this: that they had been out of work for several weeks, and were begging in the hope of getting a little food for their mother, who, they said, was dying. The detective accompanied them home, and found their statements perfectly true. The mother was evidently very ill, and there was no food in the house. The three persons had lived for four days on a single loaf of bread. According to all appearance the family was respectable, and one of the sons was in possession of testimonials accrediting him with an excellent character. The detective immediately sent for food to relieve the immediate wants, for which act the magistrate justly praised him. If all officers would take the same trouble in investigating facts and helping real distress, they would become true helpers, not merely of the sufferers, but of those charitable souls who would now gladly help the needy, but refrain on account of the oft-repeated warning that they only succeed in encouraging the idle and unworthy.

At a meeting of the subscribers of the Brechin Infirmary, held on the 12th inst., a report was read, which commenced with a mention of the great loss the infirmary had sustained in the deaths of the Earl and Countess of Dalhousie. Lord Dalhousie held the position of president of the institution for seven years, and both he and Lady Dalhousie took the greatest interest in the infirmary, and subscribed liberally to its funds.

"E. D. J." writes: "Seeing a quotation in this week's Hospital (from the *City Press*) regarding 'trained male nurses' as not coming up to the 'standard' of the opposite sex in tact, patience, or endurance, I beg to offer an indignant protest, having been myself a male nurse for three years. I have never lost one case through the want of any of the above talents. I have nursed under some of the first medical men of the day, and given every satisfaction both to them and patients (in most difficult surgical cases). I do everything for the patient, even to bed-making and mending of clothes, or darning of socks, etc.; by this means I have always dispensed with the help of a female in every way. I am anxious to defend my profession as male nurse."

We have not space this week to give an account of the discussion on Dr. Steele's paper on the "Registration of Nurses." There were upwards of 160 present, most of whom were members of the nursing profession. The majority took no part in the voting, which custom would have confined to members of the Hospitals Association only. In the result, an amendment, moved by Mrs. Bedford Fenwick to the effect that the question of registration should be submitted direct to the Medical Council, and not through the Hospitals Association, which had been carried by the casting vote of the chairman, was disposed of by the adoption of the following further amendment, moved by Dr. Steele, and carried by a large majority: "That this meeting, composed of representatives from various nurse-training schools, requests the Council of the Hospitals Association to submit a scheme for the registration of qualified nurses to the General Medical Council of Education and Registration for the United Kingdom, with a view of having a Register maintained under its auspices, and in the event of the Medical Council not acceding to this request, that the Council of the Association place itself in communication with the authorities of the various nurse-training schools, in order to frame a system of registration which would prove acceptable to the greatest number, such scheme being submitted to a meeting of a similar character."

In speaking of the Victoria Hospital for Children, Queen's Road, Chelsea, H.R.H. the Prince of Wales defined it "as good and excellent an institution as exists in the land." Next year will be noteworthy from the fact that it will include the silver wedding of their Royal Highnesses the Prince and Princess of Wales. These two facts make it singularly appropriate that the Victoria Hospital for Children should hold a grand fête and festival, to be called "The Silver Fête," in aid of its funds, which the board intend to make one of the leading events of the London season of 1888. It is intended to hold the fête in June, and Her Royal Highness Princess Louise (the patroness) has graciously intimated her intention of giving it her countenance and presence. The Board will be grateful to any lady or gentleman interested in hospitals who will send their names to be included in the list of patrons and patronesses. The only liability incurred by a steward is that of subscribing one guinea to the special fund, in return for which a card of admission to the fête each day will be issued. We hope that many of our readers will send their names without delay to Captain W. C. Blount, R.N., the courteous secretary.

